# Cardiovascular Adaptive Homeostasis in Exercise

**DOI:** 10.3389/fphys.2018.00369

**Published:** 2018-05-01

**Authors:** Kelvin J. A. Davies

**Affiliations:** ^1^Leonard Davis School of Gerontology of the Ethel Percy Andrus Gerontology Center, University of Southern California, Los Angeles, CA, United States; ^2^Molecular and Computational Biology Program, Department of Biological Sciences, Dornsife College of Letters, Arts, and Sciences, University of Southern California, Los Angeles, CA, United States; ^3^Department of Biochemistry and Molecular Medicine, USC Keck School of Medicine, University of Southern California, Los Angeles, CA, United States

**Keywords:** adaptive homeostasis, exercise, cardiovascular system, redox regulation, signal transduction, Nrf2, mitochondria, free radicals

## Abstract

Adaptive Homeostasis has been defined as, “The transient expansion or contraction of the homeostatic range in response to exposure to sub-toxic, non-damaging, signaling molecules or events, or the removal or cessation of such molecules or events.” (Davies, 2016). I propose that one of the most significant examples of adaptive homeostasis may be the adaptation of the cardiovascular system to exercise training. In particular, endurance type training involves the generation of increased levels of free radicals such as ubisemiquinone, superoxide, nitric oxide, and other (non-radical) reactive oxygen species such as hydrogen peroxide (H_2_O_2_), in a repetitive manner, typically several times per week. As long as the training intensity and duration are sub-maximal and not exhaustive these reactive species do not cause damage, but rather activate signal transduction pathways to induce mitochondrial biogenesis—the foundation of increased exercise endurance. Particularly important are the NFκB and Nrf2 signal transduction pathways which respond to reactive oxygen and nitrogen species generated during exercise. As with other examples of adaptive homeostasis the effects are transient, lasting only as long as the training is maintained. Unfortunately, the ability to adapt to exercise training declines with age, perhaps as a result of impaired Nrf2 and NFκB signaling, as does adaptive homeostasis capacity in general. Since this is an Hypothesis/Theory Paper and not a review, I have not tried to provide a comprehensive discussion of all the literature relating to exercise adaptation and the cardiovascular system. Rather, I have attempted to develop the Hypothesis or Theory that adaptive homeostasis is the foundation for adaptation of the cardiovascular system to exercise training, largely based on work from my own laboratory, that of close collaborators, and that of key contributors over a period of almost 40 years.

## Introduction

Adaptive Homeostasis has been defined as, “The transient expansion or contraction of the homeostatic range in response to exposure to sub-toxic, non-damaging, signaling molecules or events, or the removal or cessation of such molecules or events.” (Davies, 2016). Adaptive Homeostasis applies to the expansion or contraction of the homeostatic range, for any given physiological parameter, including heart rate, blood pressure, cardiac stroke volume or output, respiratory rate and volume, etc. Although Adaptive Homeostasis pertains to any signaling event that can regulate multiple physiological functions, such as heat, cold, osmotic stress, acid/base changes, nutrients, etc. the concept was actually first described (Davies, 2016) and subsequently further developed (Davies et al., 2017; Lomeli et al., 2017; Pomatto and Davies, 2017; Pomatto et al., 2017a,b,c, 2018a; 2018b; Walker et al., 2018; Zhou et al., 2018) as a function of oxidation/reduction (“Redox”) signaling. Furthermore, the “seeds” for the whole concept of adaptive homeostasis originate with studies of cardiovascular adaptation to exercise (e.g., Davies et al., 1981, 1982a,b,c, 1984). Thus, Adaptive Homeostasis would appear to be an especially good fit for this Special Research Topic on “Redox and Nitrosative Signaling in Cardiovascular System: from Physiological Response to Disease.”

Recently, we published more generalized treatises on the contribution of diminished adaptive homeostasis to multiple age-related diseases, including cardiovascular disorders (Davies et al., 2017; Lomeli et al., 2017; Pomatto and Davies, 2017), but here I will attempt to target the cardiovascular system, and its responses to exercise training, specifically. Please note that this is an Hypothesis/Theory Paper and not a review. Therefore, I have not tried to provide a comprehensive discussion of all the literature relating to exercise adaptation and the cardiovascular system, and I apologize to all those whose important work has been omitted. Rather, I have attempted to develop the Hypothesis or Theory, largely based on work from my laboratory and that of close collaborators over a period of almost 40 years, that adaptive homeostasis is the basis for adaptation of the cardiovascular system to exercise training.

## Differentiating endurance and sprint training adaptations

One of the first studied cardiovascular signaling responses is the exercise training effect. Exercise training also represents one of the most widely experienced cardiovascular adaptations affecting humans. In early studies, Holloszy et al. (Holloszy, 1967, 2008; Terjung et al., 1973) had demonstrated significant cardiovascular and skeletal muscle adaptations to endurance exercise training. In a series of studies in the late 1970's and early 1980's we set about trying to differentiate the very different adaptive responses to physical exercise training of an endurance type, vs. sprint or strength training (Davies et al., 1981, 1982b). We also sought to determine the mechanism(s) underlying exercise training adaptations although, at that time, little was known about biological signal transduction pathways. We used young male Sprague-Dawley laboratory rats to study adaptation to endurance training [10 weeks of daily treadmill running, for 5 days per week at a speed of 26.8 m/min (1.0 mph), and a slope of 8.5° (15% grade)]. Initially the rats ran for only 20 min but this was gradually increased each day such that, by week 5 (and for the next 5 weeks) the rats each ran for 120 min per day. This training protocol caused only a small (14%) increase in VO_2max_ (maximum amount of oxygen that an animal can utilize during intense, or maximal exercise) but increased endurance capacity (run time to exhaustion in a treadmill run at a constant, sub-VO_2max_ work load) four-fold and greatly increased (actually doubled) the mitochondrial content of skeletal muscles (Davies et al., 1981).

Unlike the program of endurance training described above, a regimen of sprint training (lasting 4 weeks) did not result in any significant increases in muscle mitochondrial oxidase capacity, or in total muscle mitochondrial mass, and only increased VO_2max_ by a modest 15% (Davies et al., 1982b). The sprint training program did, however, increase the capacity for high intensity workload, also called VO_2max_ work load (maximal treadmill speed at 15% grade) by 25%. The protocol used for sprint training, over a 4 week period, involved starting each session with a 2-min “warm-up” at a treadmill speed of 26.8 m/min, and then gradually increasing treadmill speed during the third minute to 97 m/min. The rats were then required to complete two 1-min periods of sprinting at 97 m/min each day (with a 10 s rest in between). To ensure that they were acclimatized to running on the treadmill (but not actually trained) control rats ran for 5 min at 26.8 m/min, twice per week. Despite the increases in VO_2max_ work load resulting from sprint training, there was no increase in endurance capacity and (as already noted above) muscle mitochondrial oxidase capacity and muscle mitochondrial mass also did not change. These results were taken as evidence that muscle mitochondrial total mass and oxidase capacity are closely coupled with exercise endurance capacity, but that VO_2max_ must be determined by other factors (Davies et al., 1982b).

In fact, the above studies (Davies et al., 1981, 1982b) also suggested that VO_2max_ might be largely determined by the oxygen carrying capacity of blood and not a function limited by mitochondrial respiration. To further test these relationships, we next used three experimental interventions with the same strain of laboratory rats described above: (1) Dietary Iron deficiency and dietary repletion (Davies et al., 1982a; Maguire et al., 1982), (2) Dietary iron deficiency and blood transfusion (Davies et al., 1984), and (3) Dietary Vitamin E (α tocopherol) deficiency (Quintanilha and Davies, 1982; Quintanilha et al., 1982).

The effects of dietary iron deficiency and dietary iron repletion on exercise performance were investigated in young male Sprague-Dawley rats (Davies et al., 1982a; Maguire et al., 1982). Iron deficiency was established in young, male rats by feeding them a low-iron diet with only 2 mg iron/kg, whereas the diet fed to control animals contained 50 mg iron/kg. After 42 days, rats fed the low-iron diet had blood hemoglobin (Hb) levels of only 3.6 ± 0.5 g/dl, whereas the control rats exhibited blood Hb of 13.7 ± 0.6 g/dl. In the iron-deficient animals, we observed 60–85% lower muscle mitochondrial oxidative capacities in comparison with controls; presumably this resulted from 30% lower mitochondrial content of muscles and significantly lower mitochondrial enzyme specific activities. In the iron-deficient rats, both VO_2_ max and VO_2_ max work load were 50% lower than seen in control animals, and exercise endurance capacity was 90% lower. To reverse deficiency, the low-iron rats were next switched to the control (iron-sufficient) diet and we carefully mapped out the time course of iron repletion and the recovery of muscle mitochondrial and exercise parameters. Blood Hb was markedly in only 3 days and was accompanied by significant improvements in both VO_2max_ and VO_2max_ work load. Importantly, however, neither mitochondrial bioenergetic functions, nor the mitochondrial content of muscle, nor muscle mitochondrial oxidative capacity, exhibited significant increases before day 5 of iron repletion, and exercise endurance capacity also did not increase before day 5. From these studies of iron-deficiency and repletion we were persuaded that muscle mitochondrial oxidase capacity probably does not normally impose a limit on VO_2max_ or VO_2max_ work load. Similarly, we concluded that the oxidative capacity of muscle (i.e., functional mitochondria) probably does limit exercise endurance capacity. Parallel studies, on the same animals, revealed that the iron deficiency protocol severely depleted mitochondrial iron sulfur proteins and cytochromes, which then caused severe limitations of up to 70–80% in the electron transport capacity, and ATP production, of mitochondria (Davies et al., 1982a; Maguire et al., 1982).

Although the above studies of iron deficiency and repletion provided temporal evidence for a dissociation between muscle mitochondrial oxidative capacity and VO_2max_/VO_2max_ workload and, conversely, provided temporal evidence for a strong association between muscle mitochondrial oxidative capacity and endurance capacity and between the oxygen carrying capacity of blood and VO_2max_ workload, no causal relationships could be concluded. To attempt to delineate cause and effect we utilized dietary iron deficiency and blood transfusions using young male weanling Sprague-Dawley rats (Davies et al., 1984). Using the same iron-deficient and control diets described above, we found that only 21 days of dietary iron deficiency caused significant anemia (Hb, 3.9 vs. 14.2 g/dl in controls) and major loss of function of muscle mitochondria, such that skeletal muscle mitochondrial total oxidative capacity was as much as 90% lower than see in control rats. VO_2max_ apparently declined by 50% as a result of iron deficiency and the maximal endurance capacity (time to exhaustion in a separate treadmill run at a constant, sub-VO_2max_ work load) of iron-deficient rats was only 10% of that seen in control animals. We next took the iron deficient rats and artificially increased their blood Hb by transfusion with packed erythrocytes. Conversely, we decreased the Hb levels of control rats by withdrawing blood and transfusing them with plasma. These procedures enabled us to match the Hb of both iron-deficient and control rats at a common value of approximately 9.5 g/dl. Raising the Hb of iron-deficient animals immediately corrected their exercise VO_2max_ such that it was only 15% below control values but, importantly, we observed no improvement whatsoever in exercise endurance capacity. These interventional experiments yielded a direct demonstration that VO_2max_ and endurance capacity depend on rather different physiological functions. Based on these results, it was posited that oxygen delivery to tissues is a major determinant of VO_2max_ but, in contrast, muscle mitochondrial capacity is a major determinant of exercise endurance capacity (Davies et al., 1984). It should also be mentioned, however, that what determines VO_2max_ is still the subject of some debate, with some investigators favoring a “cardiocentric” viewpoint whereas others consider skeletal muscles as the limiting factor.

There are also some neural reflexes that regulate the cardiovascular apparatus. One of these is the so called muscle metaboreflex, which adjusts the cardiovascular response on the basis of the metabolic conditions of working muscles. It has been speculated that in some cardiovascular diseases, and in the sedentary state, detraining affects this reflex by increasing matabolite accumulation and decreasing muscle oxidative capacity. For instance, this is the so called “muscle hypothesis” of heart failure. The interaction of intrinsic muscle factors and neural signaling may, thus, represent a potential link between fatigue, cardiovascular disregulation, and the effect of training in these pathologies.

Taken together, the above studies of endurance training; sprint training; iron deficiency, dietary repletion, and blood transfusion; and vitamin E deficiency all point to the conclusion that the mitochondrial content of muscle and, of course, mitochondrial competence have a major role in determining exercise endurance capacity. Furthermore, increased mitochondrial biogenesis during endurance exercise training would seem to be a key factor in exercise adaptive homeostasis.

## Ubisemiquinone, superoxide, and hydrogen peroxide in exercise

Our exercise studies also demonstrated, for the first time, unequivocally that free radical generation in skeletal muscles and liver increases during exercise, and is especially evident during exhaustive exercise (Davies and Hochstein, 1982; Davies et al., 1982c). Previous work by Tappel et al. (Dillard et al., 1978) had suggested that exercise caused increased free radical generation that could be measured as expired pentane but this indirect methodology was criticized as being subject to large errors due to variable pentane production by gut flora and fauna, and various other investigators produced equivocal results with other measures of lipid peroxidation. Our work used the direct approach of actually measuring free radical signals by electron spin resonance spectroscopy, and demonstrated increased concentrations of mitochondrial ubisemiquinone radicals in both muscle and liver, following exercise (Davies et al., 1982c). We were actually able to identify two discrete “pools” of semi-stabilized ubisemiquinone with stability constants high enough to support the physiological significance of Peter Mitchell's “Q Cycle Hypothesis” (Davies and Hochstein, 1982). Of greater importance for the current discussion, however, is the observation that reduction of molecular oxygen to the superoxide anion radical (${\text{O}}_{2}^{•-}$) by ubisemiquinone is a major source of intracellular ${\text{O}}_{2}^{•-}$ generation, and much if not most of the ${\text{O}}_{2}^{•-}$ so generated rapidly dismutates to form hydrogen peroxide (H_2_O_2_) (Cadenas and Davies, 2000).

Following heavy exercise, we found increases of 100–200% in observable concentrations of free radicals in rat muscles and liver (Davies et al., 1982c). The free radical signals we could observe and measure via electron spin resonance spectroscopy were largely attributable to mitochondrial ubisemiquinone radicals (Davies and Hochstein, 1982). Exhaustive exercise (but not exercise at a training level) caused significant damage, including lipid peroxidation, diminished sarcoplasmic reticulum and endoplasmic reticulum integrity, and diminished respiratory control of mitochondria. In a parallel study, we found that Vitamin E deficiency, which sensitizes animals to increased free radical oxidative damage, caused a similar damage profile (in membranes, sarcoplasmic/endoplasmic reticulum, and mitochondria) even without making the animals exercise (Davies et al., 1982c; Quintanilha and Davies, 1982; Quintanilha et al., 1982). Our earlier studies indicated that endurance capacity in exercise is principally governed by the functional muscle mitochondrial mass (Davies et al., 1981, 1982a,b, 1984), and when we exercise tested the vitamin E deficient animals (with their impaired muscle mitochondria), we found that their endurance was indeed 40% lower than that of controls (Davies et al., 1982c). These findings further strengthened our interpretation that exercise training of a serious but sub-maximal nature slightly increases free radical (ubisemiquinone and ${\text{O}}_{2}^{•-}$) and H_2_O_2_ generation, and that this is an important component of exercise adaptation. In contrast, maximal exercise to exhaustion increases free radical and H_2_O_2_ generation to dangerously high levels that can cause significant cellular damage.

Interestingly, our studies showed that the generation of ${\text{O}}_{2}^{•-}$ by purified mitochondria studied *in vitro*, increased as a function of temperature (Salo et al., 1991). When we exposed mitochondria to temperatures seen in exercising human beings (Brooks et al., 1971a,b) we found that temperature-induced partial uncoupling of oxygen consumption and ATP production accounted for the increased ${\text{O}}_{2}^{•-}$ generation (Salo et al., 1991). Thus, it is possible that temperature-induced fluctuations in the fidelity with which ubisemiquinone, and other electron carriers, such as complexes I and III, can direct electrons along the mitochondrial respiratory chain, resulting in a “leakage” of electrons directly to oxygen, may account for both significant ${\text{O}}_{2}^{•-}$ generation in exercise and a limit to exercise capacity.

Of course, today everyone recognizes both ${\text{O}}_{2}^{•-}$ and H_2_O_2_ as major intracellular signaling molecules. In 1983, however, when we first demonstrated ubisemiquinone radicals in exercise, such relationships were not understood. Despite this lack of mechanistic understanding, we wrote, “It is tempting to propose that exercise induced free radicals ………. may be the initiating stimulus to mitochondrial biogenesis.” (Davies et al., 1982c). Viewed with the hindsight of current knowledge, the results suggest that increased concentrations of ubisemiquinone radicals, and the ${\text{O}}_{2}^{•-}$ and H_2_O_2_ generated by interaction of ubisemiquinone (and/or complexes I and III) with molecular oxygen may provide a stimulus to the adaptive homeostasis, including the mitochondrial biogenesis, which results from endurance training.

## Exercise-induced generation of reactive oxygen/nitrogen species by xanthine oxidase and by neutrophils and other phagocytic cells

Since our original publications, several groups have verified increased generation of free radicals, and other reactive oxygen/nitrogen species in exercise (e.g., see Packer et al., 2008; Sachdev and Davies, 2008). In addition to the ${\text{O}}_{2}^{•-}$ and H_2_O_2_ generated by ubisemiquinone, as described above, other investigators have also described additional mitochondrial and extra-mitochondrial sources of reactive oxygen/nitrogen species in exercise (reviewed in Sachdev and Davies, 2008).

Xanthine oxidase is one such source of exercise-induced ${\text{O}}_{2}^{•-}$ and H_2_O_2_ generation, as described by Viña et al. (Heunks et al., 1999; Viña et al., 2000). These investigators noted that xanthine dehydrogenase could be converted to xanthine oxidase during intensive exercise, perhaps due to transient hypoxia. The xanthine oxidase so produced may then generate ${\text{O}}_{2}^{•-}$ and H_2_O_2_ rather than the NADH generated by its parent xanthine dehydrogenase. Importantly, the xanthine oxidase inhibitor allopurinol blocked much of the oxidation seen in tissues from intensively exercised animals.

McArdle et al. (1999) proposed that that exhaustive, eccentric, or prolonged exercise may increase generation of reactive oxygen/nitrogen species as a result of neutrophils, as well as other phagocytic type cells, overwhelming antioxidant defenses as part of an intensified immune response to cellular injury. For example, McArdle et al. (1999) reported increased concentrations of oxidized glutathione in extensor digitorum longus (EDL) muscles 3 days after an extensive program of injury-inducing contractions, although no significant glutathione oxidation was actually evident a few hours following exercise. Other investigators have also reported neutrophil/phagocyte activation and reactive oxygen/nitrogen species generation with very long duration, extreme intensity, or exhaustive exercise (Singh et al., 1994; Wareski et al., 2000; Quindry et al., 2003; Morozov et al., 2006).

Part of the problem with whole animal (or human) studies of reactive oxygen/nitrogen species, especially in exercise, is the sheer difficulty of making accurate measurements of species that are, by their very nature, short lived in biological systems. After reviewing the literature on generation of such species by mitochondrial ubisemiquinone (and/or mitochondrial complexes I and III), xanthine oxidase, neutrophils and other phagocytes, and various ill-defined sources, I think there are some valuable conclusions we may draw. First, it seems clear that there can be multiple sources of ${\text{O}}_{2}^{•-}$, H_2_O_2_, and other reactive oxygen/nitrogen species in exercise. Second, there is clearly a major difference between endurance exercise and sprint or strength training. Third, both the intensity and the duration of an exercise session affect the generation of reactive oxygen/nitrogen species. Fourth, generation of reactive oxygen/nitrogen species at low levels during non-exhaustive exercise (e.g., daily training sessions) appears to be involved in mitochondrial biogenesis whereas very high levels of reactive oxygen/nitrogen species generation during exhaustive exercise appear to contribute to tissue injury.

## Shock and stress proteins in exercise adaptation

Very heavy or exhaustive exercise induces heat-shock (in humans involving muscle temperatures that can reach 45°C, and human core body temperatures as high as 44°C), oxidative stress (as evidenced by increased levels of ${\text{O}}_{2}^{•-}$ and H_2_O_2_), and tissue damage, whereas daily exercise training at much lower intensity and duration promotes mitochondrial biogenesis (100–200% increases muscle mitochondrial mass). Following such intense and exhaustive exercise in rats, the levels of more than 15 heat shock or oxidative stress proteins, including HSP70, were elevated in skeletal muscle, heart, and liver tissues (Salo et al., 1991). Different patterns of protein transcriptional responses (incorporation of [^3^H]leucine into newly-synthesized proteins) were observed in soleus, plantaris, and EDL muscles, probably reflecting differential involvement in the exercise session and/or differential responses to heat shock and oxidative stress. Heart, liver, and skeletal muscles also exhibited diverse patterns of responses to heat shock *in vitro*, nevertheless increased levels of some five proteins, in particular HSP70 and an unidentified 106 kDa protein, were common findings.

Focusing more on HSP70, we found that mRNA levels (as measured by Northern blot with a [^32^P]-labeled *HSP70* cDNA probe), were significantly elevated in both skeletal muscle and cardiac muscle following exercise, and following either both heat-shock or oxidative stress of *ex vivo* tissues exposed to oxidative stress or heat-shock *in vitro* (Salo et al., 1991). In exhaustive exercise studies the levels of *HSP70* mRNA in skeletal muscles crested some 30–60 min after the end of the exercise session, and gradually decreased thereafter such that control levels were re-established in another 5 h. HSP70 transcription and translation may thus be seen as an appropriate physiological response to both the elevated temperatures and increased oxidation typical of heavy exercise. Extreme hyperthermia in very heavy and exhaustive exercise might actually be the proximal source for increased oxidation, because we also discovered, in this same study, a temperature-dependent uncoupling of muscle mitochondria (studied *in vitro*), with concomitant increases in ${\text{O}}_{2}^{•-}$ production: the higher the temperature applied, the more ${\text{O}}_{2}^{•-}$ generation was increased (Salo et al., 1991). Both HSP70 and HSP90 play major roles in the transport of nuclear encoded polypeptides into mitochondria, since the bulk of essential mitochondrial proteins are actually encoded in the cell nucleus and transcribed on cytoplasmic ribosomes. Thus, HSP70 and HSP90 may be vital links in the molecular mechanism of exercise-induced mitochondrial biogenesis (Salo et al., 1991). As noted above, however, there are major differences between a program of daily exercise endurance training at sub-maximal intensity and duration, and a single bout of maximal and exhaustive exercise. Thus, although it is much easier to measure the greatly increased production/generation of heat-shock or oxidative-stress proteins and free radicals or related oxidants following intense exercise to exhaustion, I propose that the much smaller elevations in these same species that occur in sub-maximal exercise, as a person trains to increase endurance, are important factors in signaling for mitochondrial biogenesis.

Importantly, rats that have actually completed a successful endurance exercise training program no longer exhibit increases in any shock or stress proteins (including no change in HSP70) at exercise levels or durations that produce significant shock/stress protein expression in untrained rats (Salo et al., 1991). This result can now be seen as an early demonstration of a key aspect of adaptive homeostasis: that the normal physiological range can be temporarily expanded in response to signaling molecules or events, but will return to its basal range if signaling is stopped for a sufficient period. In this case, exercise training is the “event” which (mediated by signaling molecules such as of ${\text{O}}_{2}^{•-}$ and H_2_O_2_) temporarily expands the physiological range for exercise tolerance such that (after weeks of training) the daily exercise workout level is no longer a stress; of course, as predicted by the adaptive homeostasis theory (Davies, 2016), this will only be true for as long as exercise training is kept up, so that a return to sedentary lifestyle will cause the expanded physiological range to gradually contract back to pre-training dimensions.

Working with close collaborators at the University of Rennes in France, we have described how exhaustive exercise increases transcription/translation of the calcineurin inhibitor RCAN1–4 (Regulator of Calcineurin 1–4) in rat skeletal muscles (Emrani et al., 2015). We had previously shown that RCAN1-4 (originally called Adapt78, DSCR1, or calcipressin) is part of a repertoire of immediate oxidative stress proteins that actually helps to improve adaptation to oxidants by inhibiting calcineurin, which dephosphorylates and inactivates many other proteins required for adaptation (Crawford et al., 1997; Ermak et al., 2002; Harris et al., 2005; Davies et al., 2007).

The levels of RCAN1-4 were elevated in both EDL and gastrocnemius muscles of rats, but not in their soleus muscles, following exercise to exhaustion (Emrani et al., 2015). Importantly, as something of an internal control, calcineurin enzymatic activity declined in EDL and gastrocnemius as levels of its inhibitor RCAN1 rose, but was unchanged in soleus muscles where RCAN1 also did not increase. Unexpectedly, the expression of calcineurin protein actually decreased in EDL, gastrocnemius, and soleus for (thus far) unexplained reasons. Another indicator of oxidative stress, protein oxidation, was also elevated in EDL and gastrocnemius muscles, but not in soleus muscles. It was concluded that oxidative “signals” generated during exercise, doubtless reactive oxygen/nitrogen species, increased the expression of RCAN1-4 protein in EDL and gastrocnemius muscles. We, therefore, proposed that up-regulation of RCAN1-4 transcription/translation and thus, induction of the signal transduction pathways that it regulates, is a significant element involved in physiological adaptative homeostasis stimulated by reactive oxygen/nitrogen species generated during exercise (Emrani et al., 2015).

Exercise also modifies the level/activity of the of mitochondrial outer-membrane-associated DNA damage repair enzyme 8-oxoguanine DNA glycosylase (OGG1) in skeletal muscle (Radak et al., 2009). Following 8 weeks of a swimming endurance training program, mitochondrial levels of carbonylated proteins were decreased (compared with control animals) and nuclear OGG1 activity was increased. These effects were reversed during de-training. Interestingly, OGG1 levels in muscle were increased following exercise endurance training, and this increase was reversed to control levels by de-training. It seems possible that endurance training may actually increase the transport of OGG1 across the mitochondrial membranes and into the matrix, thus potentially increasing the capacity for OGG1-mediated repair of oxidized DNA. In contrast, the decline in muscle OGG1 levels seen with physical inactivity could actually diminish the effective transport of OGG1 into mitochondria and decrease DNA repair (Radak et al., 2009). Finally, the mitochondrial Lon protease which protects the mitochondrial matrix against accumulation of oxidatively damaged proteins by selectively degrading them (Bota and Davies, 2002; Bota et al., 2002; Pomatto et al., 2016) is significantly induced by exercise (Koltai et al., 2012).

## Signal transduction pathways regulating cardiovascular adaptative homeostasis in exercise

From the work of many laboratories it has become abundantly clear that many forms of transient adaptation are mediated by discrete signal transduction pathways. Thus, for example, the shock, stress, or adaptive proteins discussed in the section above do not just increase (or decrease) expression on their own. Instead, dedicated signal transduction pathways exist within all cells to initiate, regulate, and terminate adaptive gene expression responses. Such adaptive homeostasis related signal transduction pathways involve sensor proteins in the cytoplasm which undergo a series of complex protein–protein interactions, transport proteins for nuclear translocation, and binding to specific regulator regions of target proteins to initiate increased transcription. Similarly, inhibitory sensor proteins can regulate the systematic down-regulation of gene expression once the need for an adaptive response has passed. Several such signal transduction pathways that operate during exercise adaptation are discussed in the sub-sections below.

### The NFκB signal transduction pathway in exercise adaptation

The nuclear factor kappa-light-chain-enhancer of activated B cells, or NFκB, signal transduction pathway contributes to adaptation and protection from oxidative stress. Under non-stressful conditions, the NFκB transcription factor resides in the cytosol, where it is bound to its specific inhibitor protein, IκB (Baeuerle and Baltimore, 1988a). Following exposure to H_2_O_2_ (or some other reactive oxygen species), NFκB is translocated to the cell nucleus, where it can bind to the upstream promoter regions of multiple target genes through its DNA binding subunits, p50 and p65. Binding of p50 and p65 to target gene promoters then elicits transcriptional activation of those target genes (Baeuerle and Baltimore, 1988b; Meyer et al., 1994). The gene encoding the mitochondrial (manganese containing) form of superoxide dismutase, or Mn-SOD, contains one such upstream promoter region, and its expression is upregulated by NFκB. Binding of NFκB to the Mn-SOD gene promoter region was originally reported by Wan et al. (1994), but the finding by Hollander et al. that an exhaustive exercise session significantly increased expression of Mn-SOD did not come for several years (Hollander et al., 2001). These results, subsequently confirmed by Ji et al. (2004), provided an example that exercise can induce adaptation and increased protection through superoxide dismutase.

Gomez-Cabrera et al. (2005) examined the effect of xanthine oxidase, via the inhibitory activity of allopurinol on NFκB activation in rats, following intensive exercise to exhaustion. The investigators found that that if, the rats received a dose of allopurionol prior to exercise, they exhibited far less oxidation of glutathione (GSH oxidized to GSSG) than did rats that were exercised with no allopurionol pre-treatment. The results were interpreted to mean that normally during intense exercise to exhaustion glutathione is oxidized by H_2_O_2_ generated by xanthine oxidase but that this can be prevented by inhibiting xanthine oxidase with allopurinol. Importantly, the allopurionol-treated rats also exhibited significantly less adaptation through the NFκB pathway. For example, the activity of Mn-SOD, among that of other inducible enzymes, increased significantly in the control animals after exhaustive exercise but was induced to a far lesser extent in rats that had received the allopurinol pre-treatment. These results provided additional evidence that NFκB plays a significant role in adaptation to oxidative stress via triggering by H_2_O_2_. Indeed, it has been found that NFκB can remain bound to target genes for many hours, or even for days, after exercise (Hollander et al., 2001) presumably causing continued elevated transcription of multiple adaptive enzymes (Gomez-Cabrera et al., 2005), thereby and playing a very important role in adapting to exercise-induced oxidative damage. Because of these observations, Gomez-Cabrera et al. (2005) also suggested that if reactive oxygen species play such major roles in initiating adaptive responses (for example via NFκB) then the practice of supplementing the diets of athletes with antioxidants should probably be reconsidered, or even discarded.

### Mitochondrial biogenesis signal transduction pathways—mitochondrial transcription factor a (TFAM) and peroxisome proliferator-activated receptor-gamma coactivator 1-alpha (PGC-1α)—in exercise adaptation

Mitochondrial transcription factor A (TFAM) peroxisome proliferator-activated receptor-gamma coactivator 1-alpha (PGC-1α) are major regulators of the mitochondrial genome (Gleyzer et al., 2005; Kaasik, 2009). In contrast nuclear respiratory factors1 and 2 (abbreviated NRF-1 and NRF-2 respectively), can control expression of many nuclear genes that actually encode mitochondrial proteins. TFAM, NRF-1, and NRF-2 are, in turn, controlled by peroxisome proliferator-activated receptor-gamma coactivator 1-alpha (PGC-1α) (Olesen et al., 2010). PGC-1α induces mitochondrial biogenesis, following activation (phosphorylation) by the mitogen-activated protein kinase (p38 MAPK) or AMP-activated protein kinase (AMPK) (Holloszy, 2008; Viña et al., 2009; Olsen, 2010). Additionally it has been shown that Sirtuin1 (SIRT1), also known as NAD-dependent deacetylase sirtuin-1, catalyzes PGC-1α deacetylation, which also activates the coactivator (Wareski et al., 2000; Nemoto et al., 2005).

Researchers have found that PGC-1α is readily induced by both acute and regular exercise and plays a significant role in muscle mitochondrial biogenesis (Terada et al., 2002; Pilegaard et al., 2003; Ikeda et al., 2006; Hart et al., 2014; Marton et al., 2015). In most of these studies, SIRT1, AMPK, NRF-1, and TFAM also accompanies (or preceded) mitochondrial biogenesis (Koltai et al., 2017). Of particular significance is the finding that aging is associated with a significant decline in mitochondrial biogenesis, but that exercise training can partially reverse this trend (Koltai et al., 2012). Importantly, PGC-1α levels decline with age but are at least partially restored with exercise training (Koltai et al., 2012). Also at least partially restored to “youthful” levels by exercise training in this study were SIRT1, AMPK, NRF-1, and TFAM, as well as actual markers of increased mitochondrial biogenesis.

### The nuclear factor erythroid-derived 2-related factor 2 (Nrf2) signal transduction pathway in exercise adaptation

The Keap1-Nrf2 signal transduction pathway is vitally important in regulating cellular and organismal adaptation to reactive oxygen and nitrogen species. This relationship is a widely observed biological phenomenon that has been verified (with Nrf2 orthologs) in numerous eukaryotic systems, including yeast, various mammalian/human cells, *Caenorhabditis elegans* nematode worms, *Drosophila melanogaster* flies, mice, rats, and human beings (Pickering et al., 2012; Zhang et al., 2015; Davies et al., 2017; Pomatto and Davies, 2017; Pomatto et al., 2017c, 2018a,b; Raynes et al., 2017). In mammals, Nrf2 is found in the cytoplasm where it forms part of a complex with Keap1 and several other proteins. The Cullin3 (Cul3) component of the Keap1-Nrf2 complex is actually an E3-ubiquitin ligase enzyme that rapidly polyubiquitinylates Nrf2 thus causing its degradation by the 26S proteasome. Since Nrf2 is synthesized at high levels, this process ensures that cytoplasmic Nrf2 undergoes constant and rapid turnover.

Upon exposure to oxidants or electrophiles, Nrf2 detaches from the Keap1 (Kelch-like ECH-associated protein 1 complex, avoids polyubiquitinylation, and instead undergoes phosphorylation by the serine/threonine kinase Akt (the RAC-alpha serine/threonine-protein kinase encoded by the *AKT* gene, also known as protein kinase B, or PKB) and protein kinase C gamma (PKC γ). Phosphorylated Nrf2 is then transported into the cell nucleus where it can bind to the upstream electrophile response elements or EPRE's (also called antioxidant response elements, or ARE's) of hundreds of target genes involved in cellular protection and adaptive homeostasis. Binding of Nrf2 to a target gene's EPRE/ARE causes increased transcription and translation, and elevated levels of the protective/adaptive protein encoded by that gene. Activation of the Nrf2 signal transduction pathway is a key, required, element for many forms of effective adaptive homeostasis, and inhibiting or blocking activation also forstalls adaptation. Nuclear factor erythroid-derived 2-related factor 1 (sometimes written as Nrf1, but which has an official symbol of NFE2L1) often binds to EPRE/ARE sites where it only poorly induces transcription and is, thus often seen as an inhibitor of Nrf2 responses (although NFE2L1 has its own target genes as well). In addition, both the transcription regulator protein Bach1 and the oncogene cMyc (Myelocytomatosis oncogene cellular homolog) appear to act as true physiological inhibitors of Nrf2 signaling (by different mechanisms) and both increase with age (Zhang et al., 2015; Zhou et al., 2018); this finding may go some way to explaining the loss of Nrf2 signaling responsiveness with aging.

If any tissue might be expected to undergo adaptive homeostasis in response to exercise training it would be skeletal muscle, and careful studies indicate that this is actually the case (Davies et al., 1981, 1982a,b, 1984). A strong link between Nrf2 signaling and mitochondrial biogenesis in exercise was reported by Merry and Ristow (2016). These authors reported that reactive oxygen and nitrogen species activate Nrf2 signaling in exercise, and work through induction of NRF-1 and TFAM to induce mitochondrial biogenesis and production of antioxidant enzymes in skeletal muscles. The authors further reported that mice with impaired Nrf2 signaling were unable to increase mitochondrial mass, endurance capacity, or whole body energy expenditure to the same extent as wild-type mice, following a 5 week program of treadmill endurance training. The duration and intensity of exercise also clearly affects Nrf2 signaling responses, with longer and more intense bouts of exercise being more effective (Li et al., 2015).

Even though the myocardium is constantly at work throughout life, Muthusamy et al. (2012) found that a session of acute endurance exercise resulted in Nrf2 signaling, with resultant enhancement of several antioxidant protective systems and defense pathways in the heart muscle of wild-type mice. Importantly, this protective example of adaptive homeostasis was not seen in Nrf2 double mutant mice. In a study published in the same year, Gounder et al. (2012) reported that Nrf2 signaling is normally impaired in the hearts of aged mice (and humans). They found that aged mice (>23 months) exhibited multiple signs of significant cardiac oxidative stress following a single bout of high-intensity endurance-type exercise, in comparison with young mice (~2 months). Furthermore, the aged mice failed to elicit a significant Nrf2 response to the endurance test. In a study of human males, Done et al. (2016) found that a single bout of cycling exercise elicited substantial Nrf2-dependent gene expression in peripheral blood mononuclear cells collected from young (23 ± 1 years) men, whereas the Nrf2 responses of aged (63 ± 1 years) men were significantly blunted. Providing more hope for the future, however, Gounder et al. (2012) found that a 6 week program of daily exercise training at more moderate levels was successful in eliciting an Nrf2-dependent adaptive response. This result raises the hope that older individuals may still be able to reap the benefits of Nrf2-dependent adaptive homeostasis, as long as the exercise stimulus is not too intense or exhaustive.

Another tissue/organ that might not have been expected to exhibit exercise adaptations is the brain. Nevertheless, Tsou et al. (2015) reported that 4 weeks of treadmill exercise training effectively diminished several of the negative neuronal effects of 1-methyl-4-phenylpyridine (MPP+) in a rodent model of Parkinson disease. The authors implicated Nrf2 signaling in their positive results, and further found that Nrf2 knock-down, using a lentivirus-carried shNrf2 delivery system, blocked the protective effects of exercise training. These results, and those above, indicate that Nrf2 has a widespread systemic role in exercise adaptation, including mitochondrial biogenesis. More in-depth reviews of the role(s) of Nrf2 in exercise adaptive homeostasis have recently been published (Done and Traustadóttir, 2016; Madhusudhanan and Rajasekaran, 2016).

## Conclusions

**Figure 1:**
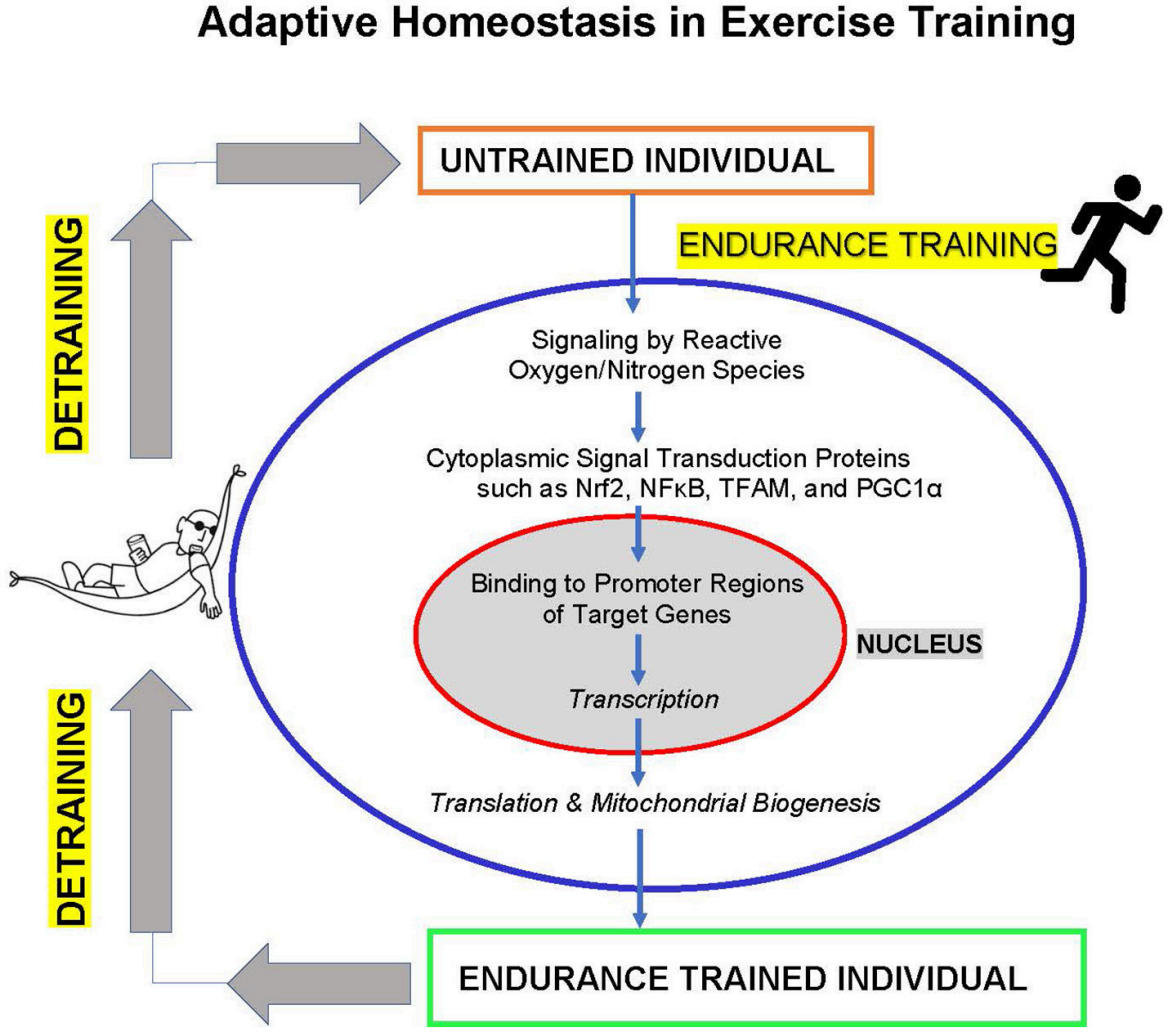
Adaptive homeostasis in exercise training. Reactive oxygen and nitrogen species such as ${\text{O}}_{2}^{•-}$, H_2_O_2_, and NO^•^ activate cytoplasmic signaling proteins such as Nrf2, NFκB, TFAM, and PGC1α to translocate to the cell nucleus where they bind to upstream promoter regions of hundreds of target genes to initiate transcription and (ultimately) translation. The protein products of these target genes include mitochondrial proteins encoded in the nucleus, leading to mitochondria biogenesis, and numerous other adaptive and protective proteins. These changes in gene expression and increased mitochondrial biogenesis (in addition to other metabolic alterations) lead to increased exercise endurance capacity. The training effect is a transient process of expanding the range of adaptive homeostasis and it only lasts as long as the training stimulus is maintained. If training is discontinued then detraining results in adaptive homeostasis returning the range of endurance capacity to pre-training levels.

The studies discussed above show that endurance exercise training involves significant adaptive homeostasis of the cardiovascular system, including extensive biogenesis of mitochondria. In fact, muscle mitochondrial oxidative capacity can now be seen as a major determinant of endurance exercise capability, and types of training that do not increase mitochondrial biogenesis (e.g., sprint training) also do not increase endurance capacity. Furthermore, impairment of mitochondrial functionality and capacity (e.g., by iron deficiency or vitamin E deficiency) imposes major limitations to exercise endurance capacity (Figure 1).

Adaptive homeostasis during exercise training is mediated by discrete signal transduction pathways operated by signaling proteins such as NFκB, TFAM, PGC-1α, Nrf2, and others. Redox regulation of signal transduction pathways by reactive oxygen species such as ${\text{O}}_{2}^{•-}$ and H_2_O_2_, and reactive nitrogen species such as NO^•^ (the nitric oxide radical), is a key factor in inducing mitochondrial biogenesis and increased cellular damage protection. Importantly, effective redox signaling, mitochondrial biogenesis, and increasing endurance capacity come as a result of sub-maximal intensity/duration exercise bouts. In other words, low levels of reactive oxygen/nitrogen species generated during serious but sub-maximal exercise training sessions are effective in recruiting signal transduction pathways to induce mitochondrial biogenesis. In contrast, exercise sessions involving maximal intensity or maximal endurance (e.g., exercise to exhaustion) generate much higher levels of reactive oxygen and nitrogen species that cause cellular damage and actually diminish effective adaptation. Thus, damage is not a necessary, or desirable, component of exercise adaptive homeostasis in the cardiovascular system.

Finally, the ability of the cardiovascular system to adapt to exercise training diminishes with age. Obviously, many factors are involved in producing the “aging phenotype,” and there are clearly many components that contribute to age-related declines, but one important factor appears to be progressively ineffective redox signal transduction and mitochondrial biogenesis with advancing age. In this regard, increasing levels of the Nrf2 inhibitors Bach1 and cMyc, that are observed with advancing age, may contribute to an age-dependent compromise of exercise adaptive homeostasis in the cardiovascular system. On a more positive note, with which to end, Kwak (2013) found that although aging is typically associated with a gradual decline in cardiac function, *“….exercise training not only improves cardiac function but also decreases the risk of heart disease.”* Specifically, Kwak found that chronic exercise training effectively diminished the age-associated mitochondrial-mediated apoptosis in the aging heart (reviewed in Kwak, 2013), giving hope that alternative pathways or mechanisms may exist to mediate the beneficial effects of exercise in older individuals.

## Author contributions

The author confirms being the sole contributor of this work and approved it for publication.

### Conflict of interest statement

The author declares that the research was conducted in the absence of any commercial or financial relationships that could be construed as a potential conflict of interest.

## Abbreviations

Nrf2: nuclear factor erythroid-derived 2-related factor 2
NFκB: nuclear factor kappa-light-chain-enhancer of activated B cells
VO_2max_: maximum amount of oxygen that an individual can utilize during intense or maximal exercise
Hb: hemoglobin
${\text{O}}_{2}^{•-}$: superoxide anion radical
H_2_O_2_: hydrogen peroxide
NO^•^: nitric oxide radical
H_2_O_2_: hydrogen peroxide
EDL: extensor digitorum longus muscle
RCAN1-4: regulator of calcineurin protein 1-4
HSP70: heat shock protein 70
OGG1: DNA damage repair enzyme 8-oxoguanine-DNA glycosylase
MnSOD: manganese superoxide dismutase (mitochondrial)
GSH: reduced glutathione
GSSG: oxidized glutathione
TFAM: mitochondrial transcription factor A
PGC-1α: peroxisome proliferator-activated receptor-gamma coactivator 1-alpha PGC-1α
NRF-1: nuclear respiratory factor 1
NRF-2: nuclear respiratory factor 2
P38MAPK: mitogen-activated protein kinase A
AMPK: AMP-activated protein kinase
SIRT1: Sirtuin1
Keap1: Kelch-like ECH-associated protein 1
Cul3: Cullin3
Akt: the RAC-alpha serine/threonine-protein kinase encoded by the *AKT* gene (also known as protein kinase B or PKB)
PKC γ: protein kinase C gamma
EPRE or ARE: electrophile response element or antioxidant response element
NFE2L1 or Nrf1: Nuclear factor erythroid-derived 2-related factor 1 (sometimes written as Nrf1 but which has an official symbol of NFE2L1)
Bach1: transcription regulator protein Bach1
cMyc: Myelocytomatosis oncogene cellular homolog
MPP+: 1-methyl-4-phenylpyridine.

## References

[B1] Baeuerle P. A. Baltimore D. (1988a). Activation of DNA-binding activity in an apparently cytoplasmic precursor of the NF-kappa B transcription factor. Cell 53, 211–217. 10.1016/0092-8674(88)90382-0 3129195

[B2] Baeuerle P. A. Baltimore D. (1988b). I kappa B: a specific inhibitor of the NF-kappa B transcription factor. Science 242, 540–546. 3140380 10.1126/science.3140380

[B3] Bota D. A. van Remmen H. Davies K. J. A. (2002). Modulation of Lon protease activity and aconitase turnover with aging and oxidative stress. FEBS Lett. 532, 103–106 10.1016/S0014-5793(02)03638-4 12459471

[B4] Bota D. Davies K. J. A. (2002). Lon protease preferentially degrades oxidized mitochondrial aconitase by an ATP-stimulated mechanism. Nat. Cell Biol. 4, 674–680. 10.1038/ncb836 12198491

[B5] Brooks G. A. Hittelman K. J. Faulkner J. A. Beyer R. E. (1971a). Temperature, skeletal muscle mitochondrial functions, and oxygen debt. Am. J. Physiol. 220, 1053–1059. 4323901 10.1152/ajplegacy.1971.220.4.1053

[B6] Brooks G. A. Hittelman K. J. Faulkner J. A. Beyer R. E. (1971b). Tissue temperatures and whole-animal oxygen consumption after exercise. Am. J. Physiol. 221, 427–431. 5560291 10.1152/ajplegacy.1971.221.2.427

[B7] Cadenas E. Davies K. J. A. (2000). Mitochondrial free radical production, oxidative stress, and aging. Free Radic. Biol. Med. 29, 222–2230. 11035250 10.1016/s0891-5849(00)00317-8

[B8] Crawford D. R. Leahy K. P. Abramova N. Lan L. Wang Y. Davies K. J. A. (1997). Hamster adapt78, mRNA is a down syndrome critical region homologue that is inducible by oxidative stress. *Arch. Biochem*. Biophys. 342, 6–12. 10.1006/abbi.1997.0109 9185608

[B9] Davies K. J. A. (2016). Adaptive homeostasis. Mol. Aspects Med. 49, 1–7. 10.1016/j.mam.2016.04.007 27112802 PMC4868097

[B10] Davies J. M. S. Cillard J. Friguet B. Cadenas E. Cadet J. Cayce R.. (2017). The oxygen paradox, the French Paradox, and age-related diseases. GeroScience 39, 499–550. 10.1007/s11357-017-0002-y 29270905 PMC5745211

[B11] Davies K. J. A. Donovan C. M. Refino C. J. Brooks G. A. Packer L. Dallman P. R. (1984). Distinguishing the effects of anemia and muscle iron deficiency on exercise bioenergetics in the rat. Am. J. Physiol.: Endocrinol. Metab. 246, E535–E543. 10.1152/ajpendo.1984.246.6.E535 6742115

[B12] Davies K. J. A. Ermak G. Rothermel B. A. Pritchard M. Heitman J. Ahnn J.. (2007). Renaming the DSCR1/Adapt78 gene family as RCAN: regulators of calcineurin. FASEB J. 21, 3023–3028. 10.1096/fj.06-7246com 17595344

[B13] Davies K. J. A. Hochstein P. (1982). Ubisemiquinone radicals in liver: implications for a mitochondrial Q cycle *in vivo*. Biochem. Biophys. Res. Commun. 107, 1292-1299. 6291526 10.1016/s0006-291x(82)80138-1

[B14] Davies K. J. A. Maguire J. J. Brooks G. A. Dallman P. R. Packer L. (1982a). Muscle mitochondrial bioenergetics, oxygen supply, and work capacity during dietary iron deficiency and repletion. *Am. J*. Physiol. Endocrinol. Metab. 242, E418-E427. 10.1152/ajpendo.1982.242.6.E418 7091311

[B15] Davies K. J. A. Packer L. Brooks G. A. (1981). Biochemical adaptation of mitochondria, muscle, and whole animal respiration to endurance training. Arch. Biochem. Biophys. 209, 539–554. 7294809 10.1016/0003-9861(81)90312-x

[B16] Davies K. J. A. Packer L. Brooks G. A. (1982b). Exercise bioenergetics following sprint training. Arch. Biochem. Biophys. 215, 260-265. 7092228 10.1016/0003-9861(82)90303-4

[B17] Davies K. J. A. Quintanilha A. T. Brooks G. A. Packer L. (1982c). Free radicals and tissue damage produced by exercise. Biochem. Biophys. Res. Commun. 107, 1198-1205. 6291524 10.1016/s0006-291x(82)80124-1

[B18] Dillard C. J. Litov R. E. Savin W. M. Dumelin E. E. Tappel A. L. (1978). Effects of exercise, Vitamin, E., and ozone on pulmonary function and lipid peroxidation. J. Appl. Physiol. 45, 927–932. 730598 10.1152/jappl.1978.45.6.927

[B19] Done A. J. Traustadóttir T. (2016). Nrf2 mediates redox adaptations to exercise. Redox Biol. 10, 191–199. 10.1016/j.redox.2016.10.003 27770706 PMC5078682

[B20] Done A. J. Gage M. J. Nieto N. C. Traustadóttir T. (2016). Exercise-induced Nrf2 signaling is impaired in aging. *Free Radic. Biol*. Med. 96, 130–138. 10.1016/j.freeradbiomed.2016.04 27109910

[B21] Emrani B. R. Rébillard A. Lefeuvre L. Gratas-Delamarche A. Ermak G. Davies K. J. A.. (2015). The calcineurin antagonist, RCAN1-4 is induced by exhaustive exercise in skeletal muscle. Free Radic. Biol. Med. 87, 290–299. 10.1016/j.freeradbiomed.2015.06.023 26122706

[B22] Ermak G. Harris C. Davies K. J. A. (2002). The DSCR1 (Adapt78) Isoform 1 protein, “Calcipressin 1” inhibits calcineurin and protects against acute calcium-mediated stress damage, including transient oxidative stress. FASEB J. 16, 814–824. 10.1096/fj.01-0846com 12039863

[B23] Gleyzer N. Vercauteren K. Scarpulla R. C. (2005). Control of mitochondrial transcription specificity factors (TFB1M and TFB2M) by nuclear respiratory factors (NRF-1 and NRF-2) and PGC-1 family coactivators. Mol. Cell Biol. 25, 1354–1366. 10.1128/MCB.25.4.1354-1366.2005 15684387 PMC548005

[B24] Gomez-Cabrera M. C. Borras C. Pallardo F. V. Sastre J. Ji L. L. Viña J. (2005). Decreasing xanthine oxidase-mediated oxidative stress prevents useful cellular adaptations to exercise in rats. J. Physiol. 567, 113–120. 10.1113/jphysiol.2004.080564 15932896 PMC1474177

[B25] Gounder S. Kannan S. Devadoss D. Miller C. J. Whitehead K. S. Odelberg S. J.. (2012). Impaired transcriptional activity of Nrf2 in age-related myocardial oxidative stress is reversible by moderate exercise training. PLOS ONE 7:e45697. 10.1371/journal.pone.0045697 23029187 PMC3454427

[B26] Harris C. D. Ermak G. Davies K. J. A. (2005). Multiple roles of the DSCR1 (Adapt78 or RCAN1) gene and its protein product calcipressin 1 (or RCAN1) in disease. Cell Mol. Life Sci. 62, 2477–2486. 10.1007/s00018-005-5085-4 16231093 PMC11139107

[B27] Hart N. Sarga L. Koch L. G. Britton S. L. Ngo J. K. Davies K. J. A. (2014). Resveratrol enhances exercise-induced adaptive responses in rats selectively bred for low running performance. Dose Response 12, 57–71. 24659933 10.2203/dose-response.13-010.Radak PMC3960954

[B28] Heunks L. M. Viña J. van Herwaarden C. L. Folgering H. T. Gimeno A. Dekhuijzen P. N. (1999). Xanthine oxidase is involved in exercise-induced oxidative stress in chronic obstructive pulmonary disease. Am. J. Physiol. 277, R1697–R1704. 10.1152/ajpregu.1999.277.6.R1697 10600916

[B29] Hollander J. Fiebig R. Gore M. Ookawara T. Ohno H. Ji L. L. (2001). Superoxide dismutase gene expression is activated by a single bout of exercise in rat skeletal muscle. *Pflügers Arch. Eur. J*. Physiol. 442, 426–434. 10.1007/s004240100539 11484775

[B30] Holloszy J. O. (1967). Biochemical adaptations in muscle. effects of exercise on mitochondrial oxygen uptake and respiratory enzyme activity in skeletal muscle. J. Biol. Chem. 242, 2278–2282. 4290225

[B31] Holloszy J. O. (2008). Regulation by exercise of skeletal muscle content of mitochondria and GLUT4. J. Physiol. Pharmacol. 59(Suppl. 7), 5–18. 19258654

[B32] Ikeda S. Kawamoto H. Kasaoka K. Hitomi Y. Kizaki T. Sankai Y. (2006). Muscle type-specific response of PGC-1alpha and oxidative enzymes during voluntary wheel running in mouse skeletal muscle. Acta Physiol. 188, 217–223. 10.1111/j.1748-1716.2006.01623.x 17054661

[B33] Ji L. L. Gomez-Cabrera M. C. Steinhafel N. Viña J. (2004). Acute exercise activates nuclear factor (NF)-kappaB signaling pathway in rat skeletal muscle. FASEB J. 18, 1499–1506. 10.1096/fj.04-1846com 15466358

[B34] Kaasik A. (2009). PGC-1α and PGC-1β regulate mitochondrial density in neurons. J. Biol. Chem. 284, 21379–21385. 10.1074/jbc.M109.018911 19542216 PMC2755862

[B35] Koltai E. Bori Z. Chabert C. Dubouchaud H. Naito H. Machida S. (2017). SIRT1 may play a crucial role in overload induced hypertrophy of skeletal muscle: an hypothesis. J. Physiol. 595, 3361–3376. 10.1113/JP273774 28251652 PMC5451718

[B36] Koltai E. Hart N. Bori Z. Taylor A. W. Goto S. Ngo J. K.. (2012). Age-associated declines in mitochondrial biogenesis and protein quality control factors are minimized by exercise training. Am. J. Physiol. Regul. Integr. Comp. Physiol. 303, R127–R134. 10.1152/ajpregu.00337.2011 22573103 PMC3404634

[B37] Kwak H.-B. (2013). Effects of aging and exercise training on apoptosis in the heart. J. Exerc. Rehabil. 9, 212–219. 10.12965/jer.130002 24278863 PMC3836520

[B38] Li T. He S. Liu S. Kong Z. Wang J. Zhang Y. (2015). Effects of different exercise durations on Keap1-Nrf2-ARE pathway activation in mouse skeletal muscle. *Free Radic*. Res. 49, 1269–1274. 10.3109/10715762.2015.1066784 26118597

[B39] Lomeli N. Bota D. Davies K. J. A. (2017). The role of diminished stress resistance, and defective adaptive homeostasis in age related diseases. Clin. Sci. 131, 2573–2599. 10.1042/CS20160982 29070521

[B40] Madhusudhanan N. Rajasekaran N. S. (2016). Exercise, Nrf2 and antioxidant signaling in cardiac aging. Front. Physiol. 7:241 10.3389/fphys.2016.00241 27378947 PMC4911351

[B41] Maguire J. J. Davies K. J. A. Dallman P. R. Packer L. (1982). Effects of dietary iron deficiency on iron-sulfur proteins and bioenergetic functions of skeletal muscle mitochondria. Biochim. Biophys. Acta 679, 210-220. 7059584 10.1016/0005-2728(82)90292-4

[B42] Marton O. L. Koltai E. Takeda M. Koch L. G. Britton S. L. Davies K. J. A.. (2015). Mitochondrial biogenesis-associated factors underlie the magnitude of response to aerobic endurance training in rats. Pflügers Arch. Eur. J. Physiol. 467, 779–788. 10.1007/s00424-014-1554-7 24943897 PMC4272336

[B43] McArdle A. van der Meulen J. H. Catapano M. Symons M. C. Faulkner J. A. Jackson M. J. (1999). Free radical activity following contraction-induced injury to the extensor digitorum longus muscles of rats. *Free Radic. Biol*. Med. 26, 1085–1091. 10.1016/s0891-5849(98)00317-7 10381177

[B44] Merry T. L. Ristow M. (2016). Nuclear factor erythroid-derived 2-like 2 (NFE2L2, Nrf2) mediates exercise-induced mitochondrial biogenesis and the anti-oxidant response in mice. J. Physiol. 594, 5195–5207. 10.1113/JP271957 27094017 PMC5023720

[B45] Meyer M. Pahl H. L. Baeuerle P. A. (1994). Regulation of the transcription factors NF-kappa B and AP-1 by redox changes. Chem. Biol. Interact. 91, 91–100. 10.1016/0009-2797(94)90029-9 8194138

[B46] Morozov V. I. Tsyplenkov P. V. GoGolberg N. D. Kalinski M. I. (2006). The effects of high-intensity exercise on skeletal muscle neutrophil myeloperoxidase in untrained and trained rats. *Eur. J. Appl*. Physiol. 97, 716–722. 10.1007/s00421-006-0193-x 16791601

[B47] Muthusamy V. R. Kannan S. Sadhaasivam K. Gounder S. S. Davidson C. J. Boeheme C. (2012). Acute exercise stress activates Nrf2/ARE signaling and promotes antioxidant mechanisms in the myocardium. *Free Radic. Biol*. Med. 52, 366–376. 10.1016/j.freeradbiomed.2011.10.440 PMC3800165 22051043

[B48] Nemoto S. Fergusson M. M. Finkel T. (2005). SIRT1 functionally interacts with the metabolic regulator and transcriptional coactivator PGC-1α. J. Biol. Chem. 280, 16456–16460. 10.1074/jbc.M501485200 15716268

[B49] Olesen J. Kiilerich K. Pilegaard H. (2010). PGC-1α-mediated adaptations in skeletal muscle. Pflügers Arch. 460, 153–162. 10.1007/s00424-010-0834-0 20401754

[B50] Packer L. Cadenas E. Davies K. J. A. (2008). Free radicals and exercise: an introduction. Free Radic. Biol. Med. 44, 123–125. 10.1016/j.freeradbiomed.2007.05.031 18191747

[B51] Pickering A. M. Linder R. A. Zhang H. Forman H. J. Davies K. J. A. (2012). Nrf2-dependent induction of proteasome and Pa28αβ regulator are required for adaptation to oxidative stress. J. Biol. Chem. 287, 10021–10031. 10.1074/jbc.M111.277145 22308036 PMC3323025

[B52] Pilegaard H. Saltin B. Neufer P.D. (2003). Exercise induces transient transcriptional activation of the PGC-1α gene in human skeletal muscle. J. Physiol. 546, 851–858. 10.1113/jphysiol.2002.034850 12563009 PMC2342594

[B53] Pomatto L. C. D. Cline M. Woodward N. Morgan T. Finch C. E. Forman H. J. (2018b). Nano-particulate exposure impacts proteasome response in young and middle-aged female mice. Free Radic. Biol. Med. [Epub ahead of print]. 10.1016/j.freeradbiomed.2018.04.574 PMC5987225 29709705

[B54] Pomatto L. C. D. Tower J. Davies K. J. A. (2018a). Sexual dimorphism and aging differentially regulate adaptive homeostasis in *Drosophila melanogaster*. J. Gerontol. Ser. A. 73, 141–149. 10.1093/gerona/glx083 PMC5861879 28525535

[B55] Pomatto L. C. D. Wong S. Tower J. Davies K. J. A. (2017a). Sex-specific differences in adaptation in *D. melanogaster* wild-type strains. Arch. Biochem. Biophys. 636, 57–70. 29100984 10.1016/j.abb.2017.10.021 PMC6508965

[B56] Pomatto L. C.-D. Davies K. J. A. (2017). The decline of adaptive homeostasis in ageing. J. Physiol. 595, 7275–7309. 10.1113/JP275072 29028112 PMC5730851

[B57] Pomatto L. C.-D. Davies K. J. A. Tower J. G. (2016). The mitochondrial Lon protease is required for age-specific and sex-specific adaptation to oxidative stress. Curr. Biol. Cell Press 27, 1–15. 10.1016/j.cub.2016.10.044 27916526 PMC5224972

[B58] Pomatto L. C.-D. Wong S. Carney C. Shen B. Tower J. Davies K. J. A. (2017b). Age- and sex-specific function of the 20s proteasome and the Nrf2/CncC signal transduction pathway in oxidative stress adaptation and resistance in Drosophila melanogaster. Aging 9, 1153–1178. 10.18632/aging.101218 28373600 PMC5425120

[B59] Pomatto L. C. D. Wong S. Tower J. Davies K. J. A. (2017c). Sex-specific adaptive homeostasis in *D. melanogaster* depends on increased proteolysis by the 20S Proteasome: data-in-Brief. Data Brief. 17, 653–661. 10.1016/j.dib.2018.01.044 29552615 PMC5852260

[B60] Quindry J. C. Stone W. L. King J. Broeder C. E. (2003). The effects of acute exercise on neutrophils and plasma oxidative stress. Med. Sci. Sports Exerc. 35, 1139–1145 10.1249/01.MSS.0000074568.82597.0B 12840634

[B61] Quintanilha A. T. Davies K. J. A. (1982). Vitamin E deficiency and photosensitization of electron transport carriers in microsomes. FEBS Lett. 139, 241-244. 6804266 10.1016/0014-5793(82)80861-2

[B62] Quintanilha A. T. Packer L. Davies J. M. S. Racanelli T. L. Davies K. J. A. (1982). Membrane effects of vitamin E deficiency: bioenergetic and surface charge density studies of skeletal muscle and liver mitochondria. Ann. N. Y. Acad. Sci. 393, 32-47. 6959560 10.1111/j.1749-6632.1982.tb31230.x

[B63] Radak Z. Atalay M. Jakus J. Boldogh I. Davies K. J. A. Goto S. (2009). Exercise improves import of 8-oxoguanine DNA glycosylase into the mitochondrial matrix of skeletal muscle and enhances the relative activity. Free Radic. Biol. Med. 46, 238–243. 10.1016/j.freeradbiomed.2008.10.022 18992806 PMC3032603

[B64] Raynes R. Juarez C. Pomatto L. C. Sieburth D. Davies K. J. A. (2017). Aging and SKN-1-dependent loss of 20S proteasome adaptation to oxidative stress in *C. elegans*. J. Gerontol. A. Biol. Sci. Med. Sci. 72, 143–151. 10.1093/gerona/glw093 27341854 PMC5233911

[B65] Sachdev S. Davies K. J. A. (2008). Production, detection, and adaptive responses to free radicals in exercise. Free Radic. Biol. Med. 44, 215–223. 10.1016/j.freeradbiomed.2007.07.019 18191757

[B66] Salo D. C. Donovan C. M. Davies K. J. A. (1991). HSP70 and other possible heat shock or oxidative stress proteins are induced in skeletal muscle, heart, and liver during exercise. Free Radical Biol. Med. 11, 239–246. 10.1016/0891-5849(91)90119-N 1937141

[B67] Singh A. Failla M. L. Deuster P. A. (1994). Exercise-induced changes in immune function: effects of zinc supplementation. J. Appl. Physiol. 76, 2298–2303. 10.1152/jappl.1994.76.6.2298 7928850

[B68] Terada S. Goto M. Kato M. Kawanaka K. Shimokawa T. Tabata I. (2002). Effects of low-intensity prolonged exercise on PGC-1 mRNA expression in rat epitrochlearis muscle. Biochem. Biophys. Res. Commun. 296, 350–354. 10.1016/S0006-291X(02)00881-1 12163024

[B69] Terjung R. L. Klinkerfuss G. H. Baldwin K. M. Winder W. W. Holloszy J. O. (1973). Effect of exhausting exercise on rat heart mitochondria. Am. J. Physiol. 225, 300–305. 10.1152/ajplegacy.1973.225.2.300 4722391

[B70] Tsou Y.-H. Shi C.-T. Ching C.-H. Huang J.-Y. Jen C.J. Kuo Y.-M.. (2015). Treadmill exercise activates Nrf2 antioxidant system to protect the nigrostriatal dopaminergic neurons from MPP+ toxicity. Exp. Neurol. 263, 50–62. 10.1016/j.expneurol.2014.09.021 25286336

[B71] Viña J. Gimeno A. Sastre J. Desco C. Aseni M. Pallardo F. V. (2000). Mechanisms of free radical production in exhaustive exercise in humans and rats; role of xanthine oxidase and protection by allopurinol. IUBMB Life 49, 539–544. 10.1080/15216540050167098 11032249

[B72] Viña J. Gomez-Cabrera M. C. Borras C. Froio T. Sanchis-Gomar F. Martinez-Bello V. E.. (2009). Mitochondrial biogenesis in exercise and in ageing. Adv. Drug Deliv. Rev. 61, 1369–1374. 10.1016/j.addr.2009.06.006 19716394

[B73] Walker C. L. Pomatto L. C.-D. Tripathi D. Davies K. J. A. (2018). Redox regulation of homeostasis and proteostasis in peroxisomes. Physiol. Rev. 98, 89–115. 10.1152/physrev.00033.2016 29167332 PMC6335096

[B74] Wan X. S. Devalaraja M. N. St Clair D. K. (1994). Molecular structure and organization of the human manganese superoxide dismutase gene. DNA Cell Biol. 13, 1127–1136. 10.1089/dna.1994.13.1127 7702755

[B75] Wareski P. Vaarmann A. Choubey V. Safiulina D. Liiv J. Kuum M. (2000). Effect of exhaustive exercise on human neutrophils in athletes. Luminescence 15, 15–20. 10.1002/(SICI)1522-7243(200001/02)15:1<15::AID-BIO570>3.0.CO;2-O 10660661

[B76] Zhang H. Davies K. J. A. Forman H. J. (2015). Oxidative stress response and Nrf2 signaling in aging. Free Radic. Biol. Med. 88, 314–336. 10.1016/j.freeradbiomed.2015.05.036 26066302 PMC4628850

[B77] Zhou L. Zhang H. Davies K. J. A. Forman H. J. (2018). Aging-related decline in the induction of Nrf2-regulated antioxidant genes in human bronchial epithelial cells. Redox Biol. 14, 35–40. 10.1016/j.redox.2017.08.014 28863281 PMC5576992

